# Calcium-Mediated Abiotic Stress Signaling in Roots

**DOI:** 10.3389/fpls.2016.01296

**Published:** 2016-08-29

**Authors:** Katie A. Wilkins, Elsa Matthus, Stéphanie M. Swarbreck, Julia M. Davies

**Affiliations:** Department of Plant Sciences, University of CambridgeCambridge, UK

**Keywords:** abiotic stress, calcium, heavy metal, hypoxia, nutrition, salinity, signaling

## Abstract

Roots are subjected to a range of abiotic stresses as they forage for water and nutrients. Cytosolic free calcium is a common second messenger in the signaling of abiotic stress. In addition, roots take up calcium both as a nutrient and to stimulate exocytosis in growth. For calcium to fulfill its multiple roles must require strict spatio-temporal regulation of its uptake and efflux across the plasma membrane, its buffering in the cytosol and its sequestration or release from internal stores. This prompts the question of how specificity of signaling output can be achieved against the background of calcium’s other uses. Threats to agriculture such as salinity, water availability and hypoxia are signaled through calcium. Nutrient deficiency is also emerging as a stress that is signaled through cytosolic free calcium, with progress in potassium, nitrate and boron deficiency signaling now being made. Heavy metals have the capacity to trigger or modulate root calcium signaling depending on their dose and their capacity to catalyze production of hydroxyl radicals. Mechanical stress and cold stress can both trigger an increase in root cytosolic free calcium, with the possibility of membrane deformation playing a part in initiating the calcium signal. This review addresses progress in identifying the calcium transporting proteins (particularly channels such as annexins and cyclic nucleotide-gated channels) that effect stress-induced calcium increases in roots and explores links to reactive oxygen species, lipid signaling, and the unfolded protein response.

## Introduction

Plant roots are exposed to a variety of abiotic stresses as they navigate the soil, foraging for nutrients and water. Cytosolic free calcium ([Ca^2+^]_cyt_) is central to the response to these stresses, acting as a second messenger but also driving exocytosis (Carroll et al., 1998). Specificity of [Ca^2+^]_cyt_ signaling is determined by the amplitude and duration (and possible oscillation) of the [Ca^2+^]_cyt_ increase, often referred to as the “signature”’ (McAinsh and Pittman, 2009), that is elicited by the stimulus. This signature would be driven by the opening of plasma membrane (PM) and endomembrane Ca^2+^-permeable channels and terminated by the activity of Ca^2+^ efflux transporters in those membranes, plus Ca^2+^-binding proteins, to restore the resting [Ca^2+^]_cyt_ of 100–200 nM. Use of organelle-targeted Ca^2+^ reporting proteins has shown that the Ca^2+^ content of the endoplasmic reticulum (ER) and Golgi increases after stress-induced transient increases in [Ca^2+^]_cyt_, strongly suggesting that Ca^2+^ is sequestered there to terminate the [Ca^2+^]_cyt_ signal (Ordenes et al., 2012; Bonza et al., 2013). Transport of Ca^2+^ into organelles is catalyzed by Ca^2+^-ATPases. There are two distinct families: The Auto-inhibited Ca^2+^-ATPases, ACA (that also operate at the PM) and the ER Ca^2+^-ATPases, ECA; reviewed by Bonza et al., 2016). The lower affinity CAX (Cation/H^+^ Exchangers) appear to be restricted to endomembranes but also facilitate Ca^2+^ sequestration (Connorton et al., 2012). Changes in organelle free Ca^2+^ in roots could also play a part in signaling, most notably in the formation of symbioses and cell death (Stael et al., 2012; Zhao et al., 2013; Wagner et al., 2015). Decoding the [Ca^2+^]_cyt_ signature will be effected by specific Ca^2+^-binding proteins. Calmodulins (CaMs) and Calmodulin-like proteins (CMLs) are encoded by multi-gene families in plants. They lack kinase domains, suggesting these proteins must target others with enzymatic activity. CaMs modulate transcription by binding to Calmodulin-binding Transcription Activators (CAMTAs) (Virdi et al., 2015). Other multi-gene families are also evident for Ca^2+^-Dependent Protein Kinases (CPKs) and Calcineurin-B Like proteins (CBLs). The latter target CBL-Interacting Protein Kinases (CIPKs) to effect cellular responses (Thoday-Kennedy et al., 2015). Changes in [Ca^2+^]_cyt_ also have the potential to activate lipid signaling pathways. A somewhat forgotten aspect of Ca^2+^ signaling is the Ca^2+^ activation of members of the Phospholipase C and Phospholipase D families (Qin et al., 1997; Hunt et al., 2004; Dressler et al., 2014; Ruelland et al., 2015; Hou et al., 2016). Phospholipase C catalyses production of diacylglycerol and inositol trisphosphate (InsP_3_) while Phospholipase D catalyses production of phosphatidic acid, thus [Ca^2+^]_cyt_ would have the capacity to trigger distinct lipid signals depending on the location and Ca^2+^-sensitivity of the phospholipases. Targets of lipid signals have been reviewed recently by Hou et al. (2016).

The vast majority of [Ca^2+^]_cyt_ measurements are from *Arabidopsis thaliana* seedlings and guard cells, achieved using the luminescent Ca^2+^-interacting aequorin protein. Far fewer studies have focused specifically on roots or utilized the greater sensitivity and spatial resolution of ratiometric fluorescent dyes. The genetically encoded YC3.6 Ca^2+^ reporter is now being used for both *Arabidopsis* and rice roots (Behera et al., 2015), holding much promise for the future. It is now clear that an identical stimulus can elicit markedly different root [Ca^2+^]_cyt_ signatures depending on genus. So far, rice root [Ca^2+^]_cyt_ signals have been found to be lower in amplitude but of longer duration than those of *Arabidopsis* (Behera et al., 2015).

**FIGURE 1:**
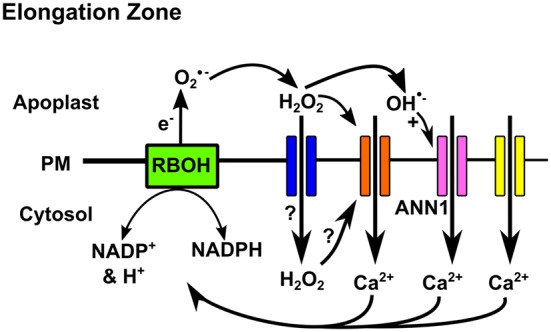
**Reactive oxygen species (ROS) regulation of PM Ca^2+^ channels in the *Arabidopsis* root elongation zone epidermis.** NADPH oxidases (RBOH) generate extracellular superoxide anion that can undergo conversion to H_2_O_2_ and hydroxyl radicals (OH^⋅^) (Richards et al., 2015). H_2_O_2_ could activate HACC (orange) at the extracellular PM face or enter the cytosol through aquaporins (blue) to activate at the cytosolic face (directly or indirectly) (Demidchik et al., 2007). Extracellular hydroxyl radicals activate Annexin 1 (pink) (Demidchik et al., 2003; Foreman et al., 2003; Laohavisit et al., 2012). Ca^2+^ influx would depolarise the PM and if unopposed this could activate DACCs (yellow) (Demidchik et al., 2002). Increased [Ca^2+^]_cyt_ could further activate RBOH.

Electrophysiological studies of root cell plasma membrane (PM) have advanced our understanding of the Ca^2+^ influx routes that could generate [Ca^2+^]_cyt_ signatures. There is a central role for PM voltage in [Ca^2+^]_cyt_ signaling, as individual stresses can hyperpolarize (render it more negative) or depolarize (render it less negative). Manipulating PM voltage elicits distinct [Ca^2+^]_cyt_ signatures and resultant transcriptional responses (Whalley et al., 2011; Whalley and Knight, 2013). Studies on root epidermal and root hair PM have shown that this membrane harbors channels that are activated by hyperpolarized voltage (Hyperpolarization-Activated Ca^2+^ Channels (HACCs); Véry and Davies, 2000; Demidchik et al., 2002, 2009; Ma et al., 2012), Depolarization-Activated Ca^2+^ Channels (DACCS); Demidchik et al., 2002; Miedema et al., 2008) and Voltage-Independent Ca^2+^ Channels (VICCs) (Demidchik et al., 2002). Thus changes in voltage would activate specific suites of channels to generate a signature. An additional tier of regulation of the PM Ca^2+^ influx routes is afforded by reactive oxygen species (ROS) that are produced during development and stress responses (**Figure 1**). This regulation depends on the specific ROS, its position, the cell type and the cell’s developmental state. In *Arabidopsis* roots, sensitivity of PM Ca^2+^ channel activation by extracellular H_2_O_2_ decreases as epidermal cells mature but is still greater than that of the cortex (Demidchik et al., 2007). A similar picture emerges for extracellular hydroxyl radicals, which elicit greater PM Ca^2+^ influx currents in the epidermis and root hairs than the pericycle (Demidchik et al., 2003; Foreman et al., 2003). In epidermal PM of the elongation zone, extracellular hydroxyl radicals elicit different Ca^2+^ channel activity to extracellular H_2_O_2_ (Demidchik et al., 2003, 2007). Thus, ROS will play a significant part of generating cell-specific [Ca^2+^]_cyt_ signatures in response to stress.

Stress-induced [Ca^2+^]_cyt_ elevation in roots remains poorly understood in terms of the genes encoding the PM or endo membrane Ca^2+^ channels involved. Plants have multi-gene families of Glutamate Receptor-Like channels (GLR; activated by a range of extracellular nitrogenous ligands) and Cyclic Nucleotide-Gated channels (CNGC; activated by intracellular cyclic nucleotides), with each gene encoding a potential subunit of a potentially tetrameric channel. Some members have been characterized as having Ca^2+^ channel forming ability (reviewed by Swarbreck et al., 2013 and Weiland et al., 2016). Membrane residency has yet to be determined for all proteins and while the majority tested are in the PM, in *Arabidopsis* GLR3.5 has been localized to both mitochondria and chloroplast, depending on its splicing variant (Teardo et al., 2015), CNGC19 to the vacuole (Yuen and Christopher, 2013) and CNGC20 potentially to both PM and vacuole (Fischer et al., 2013; Yuen and Christopher, 2013). Mechanosensitive Ca^2+^ channels of the PM have also been identified (Nakagawa et al., 2007; Hou et al., 2014; Yuan et al., 2014; Kamano et al., 2015), as has a vacuolar Ca^2+^ efflux channel TPC1 (Two Pore Channel1; Peiter et al., 2005). Root cells will express specific complements of these genes and their transcription can change under abiotic stress (Dinneny et al., 2008; Roy et al., 2008), with the implication that stress resets the [Ca^2+^]_cyt_ signaling system.

The threat of abiotic stress is global. Drought threatens plant productivity across continents, with water shortage not only imposing an osmotic challenge but also leading to soil hardness that roots must overcome. Changing weather patterns are bringing greater rainfalls to some areas (particularly Northern Europe) thus leading to the threat of hypoxic challenge from waterlogged soil (Shabala et al., 2014). Salinity stress arising from sodic soils is made worse by irrigation and counteracting nutritional deprivation by fertilizer application comes with an increasing economic and environmental cost. In this review, the effects of salinity, water availability (including soil hardness), nutritional deprivation, heavy metals and cold on root [Ca^2+^]_cyt_ will be addressed. The candidate channels for elevating [Ca^2+^]_cyt_ in roots will be introduced and the downstream consequences of the signal will be reviewed.

## Salinity Stress from Channel to Transcription

**FIGURE 2:**
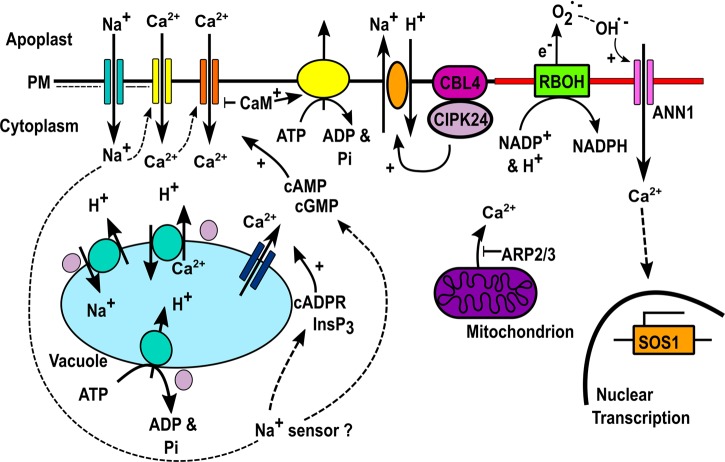
**Ca^2+^ transporters in salinity-stress signaling.** Na^+^ enters root epidermis and depolarises the PM, possibly activating DACCs (yellow) with the [Ca^2+^]_cyt_ increase also possibly activating HACCs (orange). Na^+^ sensing results in cGMP/cAMP production that could activate CNGC HACCs (Shabala et al., 2015). InsP_3_ and cADPR production causes Ca^2+^ release from stores by unknown channels (DeWald et al., 2001; Tracy et al., 2008; Zhang Y. et al., 2015; Zhang X. et al., 2015). ARP2/3 restricts mitochondrial Ca^2+^ efflux (Zhao et al., 2013). [Ca^2+^]_cyt_ activation of RBOH NADPH oxidase activity would promote extracellular hydroxyl radical formation and activation of Annexin1-mediated Ca^2+^ influx (pink) (Laohavisit et al., 2013). We hypothesize that these are held in a microdomain (red). This leads to *SOS1* transcription (Laohavisit et al., 2013). CaM activation would restrict CNGC-mediated Ca^2+^ influx and promote efflux by ACA Ca^2+^-ATPase activation (Boursiac and Harper, 2007). [Ca^2+^]_cyt_ elevation activates CBL4 and CIPK24 (purple) leading to SOS1-mediated Na^+^ extrusion (Liu et al., 2000). CIPK24 activates vacuolar V-ATPase to provide the driving force for NHX1 and CAX1 to sequester Na^+^ and Ca^2+^ respectively, also activated by CIPK24 (Cheng et al., 2004; Batelli et al., 2007; Quintero et al., 2011).

The transporters for Na^+^ influx into the root are not fully known but include the PM cyclic nucleotide-gated channels CNGC3 (Gobert et al., 2006) and CNGC10 (Guo et al., 2008; Jin et al., 2015) in *Arabidopsis*. Na^+^ ingress is opposed by the Annexin1 protein and the AGB1 heterotrimeric G protein subunit in *Arabidopsis* roots (Laohavisit et al., 2013; Yu and Assmann, 2015). The mechanisms for sensing the increase in cytosolic Na^+^ remain obscure (Maathuis, 2014; Shabala et al., 2015) however what *is* clear is that Na^+^ entry depolarizes the root epidermal PM voltage (Maathuis, 2014). This is significant in that it implicates depolarization-activated and voltage-independent PM Ca^2+^-permeable channels in generating the transient [Ca^2+^]_cyt_ increases observed in roots in response to NaCl (**Figure 2**). Both channels types are present in *Arabidopsis* root epidermal PM (Demidchik et al., 2002). However, involvement of hyperpolarization-activated PM Ca^2+^ channels should not be dismissed because increasing [Ca^2+^]_cyt_ shifts their activation voltage to more depolarized values (Véry and Davies, 2000; Demidchik et al., 2002) and they are implicated in the *Arabidopsis* root response to NaCl (Ma et al., 2012; Laohavisit et al., 2013). Critically, hyperosmotic stress hyperpolarizes root epidermal PM voltage (Maathuis, 2014) and this would potentially be a basis for generating a component of the [Ca^2+^]_cyt_ signal specific to the hyperosmotic component of NaCl stress.

### Ca^2+^ Influx across the PM

Application of NaCl can cause a heterogeneous increase in *Arabidopsis* and rice root [Ca^2+^]_cyt_ that depends on cell type, external [Ca^2+^], and the bathing medium’s effect on PM voltage (Kiegle et al., 2000; Tracy et al., 2008; Laohavisit et al., 2013; Choi et al., 2014; Zhang Y. et al., 2015). Pericycle cells of *Arabidopsis* have a lower amplitude of [Ca^2+^]_cyt_ increase and a more variable recovery phase than the surrounding tissues (Kiegle et al., 2000), suggesting a cell-specific transporter complement. Block of NaCl-induced [Ca^2+^]_cyt_ increase by lanthanides implicates PM Ca^2+^ influx channels in the response of both *Arabidopsis* and rice roots (Tracy et al., 2008; Zhang Y. et al., 2015). The anti-apoptotic protein Bcl-2 mimics lanthanides in that its overexpression impairs the NaCl-induced [Ca^2+^]_cyt_ increase in rice roots (Kim et al., 2014). Clearly, the [Ca^2+^]_cyt_ increase could lead to cell death if it were great enough and Bcl-2 may interact directly with PM channels. The genetic identities of the PM Ca^2+^ influx channels initiating the NaCl-induced [Ca^2+^]_cyt_ increase remain unknown. It has been suggested that they may be Ca^2+^-permeable CNGCs (Shabala et al., 2015) as salinity stress increased cytosolic cGMP in *Arabidopsis* seedlings within seconds (Donaldson et al., 2004). The source of cGMP may prove to be critical as only the [Ca^2+^]_cyt_ response to low NaCl (50 mM) was sensitive to inhibition of soluble guanylyl cyclase activity and the signal evoked by an equivalent osmotic stress was insensitive (Donaldson et al., 2004). It remains feasible that neither the ionic nor osmotic components of the [Ca^2+^]_cyt_ increase in response to high [NaCl] require cGMP. The osmotic component of NaCl stress could increase [Ca^2+^]_cyt_ in *Arabidopsis* roots through the PM mechanosensitive Ca^2+^-permeable channel OSCA1 (Reduced Hyperosmolality-Induced [Ca^2+^]_I_ increase1). This channel mediates the [Ca^2+^]_cyt_ response to hyperosmotic stress (Yuan et al., 2014). Its discovery through a screen of aequorin-expressing mutants (Yuan et al., 2014) highlights the potential of this approach for identification of channels (and their families) involved in stress-induced [Ca^2+^]_cyt_ elevation.

The initial increase in [Ca^2+^]_cyt_ could be amplified by the production of ROS sourced ultimately by PM NADPH oxidases (encoded by *Respiratory Burst Oxidase Homolog* genes), with [Ca^2+^]_cyt_ activating these enzymes through their EF hands (**Figure 2**). In accordance with this, *Arabidopsis* root cortical cells lacking RBOHD and F have much lower PM hyperpolarization-activated Ca^2+^ activity in response to NaCl challenge than wild type (Ma et al., 2012). Mutant seedlings have an impaired [Ca^2+^]_cyt_ response. *Arabidopsis* RBOHD can be activated by CIPK26/CBL1/9 (Drerup et al., 2013) but a direct link to stress has not been shown. Extracellular hydroxyl radicals are likely to be the ROS involved in [Ca^2+^]_cyt_ elevation (Chung et al., 2008; Demidchik et al., 2010; Laohavisit et al., 2013; Richards et al., 2015). The extracellular superoxide anions produced by NADPH oxidases are readily converted to H_2_O_2_ and wall Fe/Cu act as Fenton catalysts to generate hydroxyl radicals (Richards et al., 2015). Their production by *Arabidopsis* roots is significantly increased under NaCl stress (Demidchik et al., 2010) and RBOHC is implicated as a driver for production (Chung et al., 2008). Extracellular hydroxyl radicals activate a PM Ca^2+^ influx in *Arabidopsis* root epidermis (Demidchik et al., 2003; Foreman et al., 2003) that has now been shown to be mediated by Annexin1 (Laohavisit et al., 2012).

Annexins are Ca^2+^-binding proteins that can bind to or insert into membranes and are implicated in stress reactions (Laohavisit and Davies, 2011; Davies, 2014). Critically, when root epidermal protoplasts are challenged with NaCl, the resultant PM hyperpolarization-activated Ca^2+^ influx is lost in the *annexin1* loss of function mutant and the [Ca^2+^]_cyt_ signal is impaired (Laohavisit et al., 2013). Hydroxyl radicals are potent but short-lived so their effects must be close to the site of production (Richards et al., 2015). As NADPH oxidases can be held in lipid rafts it may be that hydroxyl radicals target co-resident channels. Finally, extracellular ATP levels of *Arabidopsis* roots increase in response to NaCl (Dark et al., 2011). Extracellular ATP activates root epidermal PM hyperpolarization-activated Ca^2+^ influx channels *via* RBOHC (Demidchik et al., 2009), suggesting the involvement of extracellular ROS in channel activation. Whether these channels involve Annexin1 remains to be determined but both the *annexin1* and *rbohc* loss of function mutants are impaired in [Ca^2+^]_cyt_-dependent transcriptional responses under NaCl stress (Chung et al., 2008; Laohavisit et al., 2013).

### Calcium Release from Stores

Although not demonstrated in roots, the *Arabidopsis* Actin-Related Protein2/3 (ARP2/3) acts to limit NaCl-induced [Ca^2+^]_cyt_ increase, partly by limiting Ca^2+^ release from mitochondria. In the *arp2/3* mutant, the [Ca^2+^]_cyt_ increase is greater than wild type and so is the extent of mitochondrial-driven cell death (Zhao et al., 2013). Release of vacuolar Ca^2+^ to the cytosol in *Arabidopsis* roots may be by a Na^+^/Ca^2+^ exchanger encoded by *AtNCL* (*Na^+^/Ca^2+^ Exchanger-Like*; Wang et al., 2012; Li P.H. et al., 2016). This tonoplast protein is thought to sequester Na^+^ into the vacuole, coupled to the release of vacuolar Ca^2+^. The concomitant increase in [Ca^2+^]_cyt_ could provide a negative feedback mechanism to limit further transport as Ca^2+^ binding to the exchanger’s EF hands has been shown to be inhibitory *in vitro* (Li P.H. et al., 2016). Pharmacological approaches have also implicated internal stores in the NaCl-induced [Ca^2+^]_cyt_ increase in roots of both *Arabidopsis* and rice. Inhibitors of store release of Ca^2+^ by cADPR (cyclic ADP ribose) and inositol trisphosphate (InsP_3_) suggested involvement of the InsP_3_ pathway in *Arabidopsis* roots (Tracy et al., 2008). Moreover, salt and hyperosmotic stress in *Arabidopsis* roots caused an InsP_3_ accumulation that correlated well with [Ca^2+^]_cyt_ increase (DeWald et al., 2001). The rice [Ca^2+^]_cyt_ signal was also sensitive to disruption of putative InsP_3_-gated store release whilst impairment by thapsigargin implicated the ER as a participating store (Zhang Y. et al., 2015). However, using ER-targeted YC3.6, Bonza et al. (2013) detected an *increase* in *Arabidopsis* root ER [Ca^2+^] in response to salt stress. This followed the salt-induced [Ca^2+^]_cyt_ increase and critically, a drop in ER [Ca^2+^] prior to the [Ca^2+^]_cyt_ increase was never observed. Therefore, with the level of resolution available, it appears that in this system the ER does not contribute to the [Ca^2+^]_cyt_ signal through store release but acts to return [Ca^2+^]_cyt_ to resting levels. Studies on *Populus euphratica* cultured cells identified the vacuole as the site of InsP_3_ and cADPR action (Zhang X. et al., 2015). Certainly, Phospholipase C isoforms (as the source of InsP_3_) are firmly implicated in salt stress responses (reviewed by Ruelland et al., 2015).

The genetic identities of the endomembrane Ca^2+^-permeable channels implicated by pharmacological studies remain elusive. GLRs have recently been postulated to be involved in ER Ca^2+^ release (Weiland et al., 2016). The TPC1 vacuolar channel of *Arabidopsis* would be capable of releasing Ca^2+^ to the cytosol and recent analyses of its crystal structure has thrown greater light on its regulation by voltage and EF hands (Guo et al., 2016; Kintzer and Stroud, 2016). At current resolution afforded by YC3.6, its loss does not appear to have a significant impact on the magnitude of the *Arabidopsis* root [Ca^2+^]_cyt_ increase to salt, rather it slightly delays the response (Choi et al., 2014). However, TPC1 has a significant part to play in the propagation of a [Ca^2+^]_cyt_ wave that travels from the root apex through the cortical and endodermal tissue to signal the NaCl challenge to the shoots and elicit a transcriptional response. ROS also relay a salinity stress signal (reliant on RBOHD) in *Arabidopsis* leaves (Miller et al., 2009) but whether this occurs in roots and is involved in propagating the [Ca^2+^]_cyt_ wave remains to be tested.

### Decoding, Na^+^ Clearance, Transcription and the Unfolded Protein Response

Salinity-induced [Ca^2+^]_cyt_ elevation in roots drives a transcriptional response (Laohavisit et al., 2013; Zhang Y. et al., 2015) and post-translational modifications. The proteins sensing the NaCl-induced [Ca^2+^]_cyt_ increase are now being elucidated. For example, the *Arabidopsis* vacuolar Two Pore K^+^ channel 1 (TPK1) would bind Ca^2+^, and open to release K^+^ to the cytosol to maintain a favorable Na^+^/K^+^ ratio (Latz et al., 2013). This could be further enhanced by phosphorylation by CPK3, which requires micromolar [Ca^2+^] for activity. CPK3 is present at both the PM and vacuole. It does not appear to contribute to a transcriptional response under salt stress but has a discrete set of protein targets to phosphorylate (Mehlmer et al., 2010). On prolonged salt stress, CPK29 expression is induced. This protein can phosphorylate TPK1 at sub-micromolar [Ca^2+^] and is envisaged to be part of longer-term K^+^ homeostasis in adapted roots (Latz et al., 2013). Also in *Arabidopsis*, CPK27 (present at the root PM) acts to promote Na^+^ efflux (Zhao et al., 2015). Intriguingly, CPK7 acts to limit water transport in *Arabidopsis* roots through lowering PIP1 aquaporin abundance (Li et al., 2015) but whether this is relevant to salinity or osmotic stress is not yet known. It can be readily envisaged that calmodulins will bind Ca^2+^ and as these are negative regulators of CNGC channels (Hua et al., 2003), would act to limit further Na^+^ or Ca^2+^ influx at the PM. Further, CaM activation of ACAs (Autoinhibited Ca^2+^ ATPases) would restore [Ca^2+^]_cyt_ to pre-stimulus levels (Boursiac and Harper, 2007). Expression of ACAs varies with salt stress and, as shown by rice roots, can relate to salt tolerance (Yamada et al., 2014).

The Salt Overly Sensitive (SOS) pathway lies downstream of the root [Ca^2+^]_cyt_ increase. Delineated in *Arabidopsis* and now acknowledged as operating in crops and trees (Thoday-Kennedy et al., 2015), the SOS pathway leads to Na^+^ efflux from the cytosol. Efflux across the PM is mediated by the SOS1 Na/H^+^ antiporter. Salt stress induction of *SOS1* transcription lies downstream of Annexin1 in *Arabidopsis* roots and as SOS1 is required for adaptive adventitious root formation, the *annexin1* loss of function mutant accordingly produces fewer of these than wild type (Laohavisit et al., 2013). Additionally, the stability of salt stress-induced *SOS1* transcript requires RBOHC (Chung et al., 2008), further suggesting that this NADPH oxidase and Annexin1 may be in the same pathway. In *Arabidopsis* roots, increased [Ca^2+^]_cyt_ is sensed at the PM by CBL4 (SOS3) which can then react with the serine/threonine protein kinase CIPK24 (SOS2) (Liu et al., 2000). The resultant CBL/CIPK complex phosphorylates the PM Na/H^+^ antiporter SOS1 at its auto-inhibitory C-terminus to achieve activation of Na^+^ efflux (Quintero et al., 2011). Activation of SOS1 can also be achieved in *Arabidopsis* by Mitogen Activated Protein Kinase6 (MPK6) and loss of MPK6 function impairs root growth under salt stress (Yu et al., 2010). MPK6 is activated by a phosphatidic acid produced under salt stress by Phospholipase Dα1 (PLDα1) (Yu et al., 2010). As this PLD isoform contains a Ca^2+^-binding C2 domain for its activation it is feasible that salt-induced [Ca^2+^]_cyt_ increase could activate SOS1 through this lipid-mediated pathway via MPK6 (Yu et al., 2015). This may help explain the importance of activation of PLDs in salt-stressed crop roots such as barley (Meringer et al., 2016). SOS2 may also promote activity of the vacuolar V-type H^+^-ATPase to provide the driving force for Na^+^ sequestration (Batelli et al., 2007). The NHX1 Na^+^-H^+^ exchanger may also be regulated by SOS2 to aid vacuolar Na^+^ sequestration (Qiu et al., 2004) and SOS2 activation of the vacuolar CAX1 Ca^2+^/H^+^ antiporter would help terminate a [Ca^2+^]_cyt_ signature through Ca^2+^ sequestration (Cheng et al., 2004). Other CAX are involved in root tolerance of salt stress and the pathways to their induction and regulation now need to be identified (Yamada et al., 2014).

Salt exposure puts the plant’s ER under stress, leading to an accumulation of unfolded or misfolded proteins that could lead to cell death (Liu et al., 2007, 2011). Such ER stress triggers upregulation of a suite of responses termed the “Unfolded Protein Response” (UPR), in which folding capacity is upregulated (including by Ca^2+^-regulated chaperones), translation is curtailed and the ER-associated degradation pathway acts to lower the aberrant protein load (Deng et al., 2013; Ruberti et al., 2015; Hossain et al., 2016; Wan and Jiang, 2016). Expression of the ER Ca^2+^-binding chaperones Calnexin and Calreticulin has been shown to upregulated in the *Arabidopsis* UPR (Christensen et al., 2008; Liu et al., 2011) and it would now be interesting to test whether these are involved in regulating levels of Ca^2+^ in the ER under stress. However, expression of rice’s only calnexin gene is decreased under salt stress (Sarwat and Naqvi, 2013). It is not yet clear whether the salt-induced [Ca^2+^]_cyt_ signal in roots (or other parts of the plant) helps initiate or regulate the UPR. It is feasible that the salt-induced [Ca^2+^]_cyt_ increase activates the root PM Phospholipase C2 (Hunt et al., 2004) because the *Arabidopsis* loss of function mutant is hypersensitive to tunicamycin, which can induce the UPR (Kanehara et al., 2015). With IP_3_ as the product of Phospholipase C activity, this would implicate IP_3_-mediated release of Ca^2+^ from stores. Indeed, for *Arabidopsis* seedlings the application of 2-aminoethoxydiphenyl borate as an inhibitor of IP_3_-mediated Ca^2+^ release prevented salt stress-induced transcription of *BIP1/2* (encoding an ER chaperone) as a diagnostic of a UPR response (Liu et al., 2011). Intriguingly, application of La^3+^ as a blocker of PM Ca^2+^ influx prevented the upregulation of UPR gene expression which was produced by application of spermine to *Arabidopsis* seedlings (Sagor et al., 2015). Exogenous spermine (as with salt) can depolarise the root epidermal PM (Pottosin et al., 2014) and can also attenuate hydroxyl radical-induced cation fluxes at root epidermal PM (Zepeda-Jazo et al., 2011). Whether spermine and salt stress share a common pathway to the UPR response merits further investigation.

## Water Availability is Signaled Through [Ca^2+^]_cyt_

The hyperosmotic challenge in [Ca^2+^]_cyt_ determinations is acute and does not mimic the chronic, progressive drought conditions that roots may face. Nevertheless, such studies have proved fruitful. As described above, the *Arabidopsis* OSCA1 PM Ca^2+^ influx channel drives the root’s initial hyperosmotic stress [Ca^2+^]_cyt_ signal (Yuan et al., 2014). In an elegant study, heterologous expression of *Arabidopsis* genes in Chinese Hamster Ovary (CHO) cells containing the Fura-2 Ca^2+^-reporting dye lead to the identification of Calcium-permeable Stress-gated cation Channel1 (CSC1; Hou et al., 2014), a close relative of OSCA1. CSC1 has been characterized in CHO cells as a PM Ca^2+^-permeable channel that is activated by hyperosmotic stress. It is resident in plant PM (Hou et al., 2014) and is expressed in roots but to date an *in planta* role remains unreported. Targeting aequorin to the cytosolic face of the *Arabidopsis* vacuolar membrane has revealed the capacity of the vacuole to release Ca^2+^ in response to acute hyperosmotic stress, with pharmacological intervention suggesting an involvement of InsP_3_ (Knight et al., 1997). As with salt stress, hyperosmotic stress-induced [Ca^2+^]_cyt_ increase could activate Phospholipases and initiate lipid signaling. Osmotic stress activates PLD in barley roots (Meringer et al., 2016). Although not tested directly, phosphatidic acid downstream of PLD could be involved in the activation of two sucrose non-fermenting-1 related protein kinase 2 proteins (SnRK2.4 and 2.10) in *Arabidopsis* roots under hyperosmotic stress. These SnRK2s are also activated under salt stress and relocate from the root epidermal cytosol to the PM; loss of function impairs root growth (McLoughlin et al., 2012). Work using Golgi-targeted aequorin in *Arabidopsis* seedlings has shown that an increase in Golgi [Ca^2+^] follows the [Ca^2+^]_cyt_ increase induced by hyperosmotic stress, suggesting that this organelle helps terminate the [Ca^2+^]_cyt_ signal (Ordenes et al., 2012).

The ABA produced under drought stress inhibits primary root growth and [Ca^2+^]_cyt_ is likely to play a role in the signaling pathway as exogenous ABA elevates root [Ca^2+^]_cyt_, which in *Arabidopsis* roots is controlled by the PM Proline-rich Extensin-like Receptor Kinase4 (PERK4) (Bai et al., 2009). PERK4’s extracellular domain is wall associated and its intracellular domain has kinase activity. PM HACCs lie downstream of ABA and PERK4. The *perk4* mutant is not only impaired in ABA-induced HACC activity and [Ca^2+^]_cyt_ activation but also in ABA inhibition of primary root elongation (Bai et al., 2009). This implies that [Ca^2+^]_cyt_ elevation acts to arrest growth. RBOHD/F may lie downstream of PERK4 and upstream of HACCs in the root ABA pathway if the HACCs were ROS-activated. The *rbohd/f* mutant is impaired in both ABA-induced HACC activation and [Ca^2+^]_cyt_ elevation (Jiao et al., 2013). Another possibility is that CNGCs are involved as ABA can increase cGMP levels (Isner et al., 2012.). GLRs are firmly implicated in the drought response; overexpression of rice GLR1 and GLR2 enhances drought tolerance of both rice and *Arabidopsis* (Lu et al., 2014). The [Ca^2+^]_cyt_ increase is likely to lead to a transcriptional response as Ca^2+^-sensing proteins are activated. ABA increases abundance and activity of *Arabidopsis* CPK4 and CPK11 leading to phosphorylation of the ABA-responsive transcription factors ABF1 and ABF4 and induction of drought stress genes (Zhu et al., 2007). Drought leads to CAMTA1 activity and the regulation of discrete gene sets including those for drought recovery (Pandey et al., 2013). In maize roots, drought stress triggers activity of CIPK8 (which interacts with CBL1, 4, and 9) and likely leads to adaptive transcription (Tai et al., 2016).

Hypoosmotic stress also elevates [Ca^2+^]_cyt_ and is relevant to waterlogged soils. In *Arabidopsis*, root PM harbors two mechanosensitive Ca^2+^-permeable channels, MCA1 and MCA2 (Mid1-Complementing Activity; Kamano et al., 2015). MCA1 responds to hypoosmotic stress to elevate [Ca^2+^]_cyt_ (Nakagawa et al., 2007). Rice only harbors an MCA1 in the PM. It is present in the root and mediates hypoosmotic shock-induced [Ca^2+^]_cyt_ expression in cultured cells, probably lying upstream of NADPH oxidase activity (Kurusu et al., 2012). Abundant water not only exposes roots to potential hypoosmotic stress but also risks limiting their oxygen supply, the consequences of which are reviewed in the following section.

## Mechanistic Basis of [Ca^2+^]_cyt_ Response to O_2_ Deficiency Remains Poorly Understood

Oxygen deficiency (hypoxia) or absence (anoxia) causes transient increases in root [Ca^2+^]_cyt_ but the signature is organ- and species-dependent (reviewed by Shabala et al., 2014). For example, when challenged by anoxia, root protoplasts from hypoxia-tolerant rice display a greater [Ca^2+^]_cyt_ signature than hypoxia-intolerant wheat root protoplasts (Yemelyanov et al., 2011). The location of the Ca^2+^ influx also varies; use of pharmacological blockers showed that the rice signature was generated by both PM influx and store release whilst wheat appeared to rely solely on stores (Yemelyanov et al., 2011). The types of Ca^2+^ channels mediating the [Ca^2+^]_cyt_ increase have yet to be identified in any species. However, those activated by PM voltage depolarization are implicated. As O_2_ deficiency lessens ATP production, activity of the PM H^+^-ATPase can be compromised thus resulting in a less negative (depolarized) PM voltage as H^+^ efflux is curtailed. The extent and duration of membrane depolarization varies with sensitivity to O_2_ deprivation and cell type. Values of -70 to -80 mV have been reported for O_2_-deprived barley root cells (Zeng et al., 2014) and theoretically these would be sufficient to activate PM depolarization-activated Ca^2+^ influx channels (Miedema et al., 2008) to contribute to an hypoxia/anoxia [Ca^2+^]_cyt_ signature. Downstream of the [Ca^2+^]_cyt_ signature, it is likely that CaM and ROPs (Rho GTPase) are activated (reviewed by Shabala et al., 2014). In *Arabidopsis* roots, hypoxia causes rapid upregulation of *CML38* expression and this protein appears to require Ca^2+^ to associate with the cytosolic stress granules that form and store messenger RNA ribonucleoproteins (Lokdarshi et al., 2016).

RBOH activity is firmly implicated in the response to low O_2_. In *Arabidopsis*, *RBOHD* expression is induced by hypoxia and is required for transcription of hypoxia-induced genes (Yang and Hong, 2015). Hypoxia also induces ethylene production and in wheat roots this causes RBOH induction (Yamauchi et al., 2014). A further level of regulation has been found in *Arabidopsis*; the Hypoxia Responsive Universal Stress Protein 1 (HRU1) interacts with GTP-bound ROP2 and RBOHD (Gonzali et al., 2015). HRU1 is one of 44 putative universal stress proteins in *Arabidopsis*. It exists as a cytosolic dimer but anoxia promotes monomer formation and increased association with the PM. There it is thought to be part of a mobile complex with ROP2 and AtRBOHD leading to activation of that NADPH oxidase (Gonzali et al., 2015). Although RBOH activity has been linked to PM Ca^2+^ channel activation in several abiotic stress scenarios, hypoxia and anoxia have yet to be tested.

Ethylene has been shown to activate PM Ca^2+^-permeable channels (with a weak voltage dependence) in tobacco suspension cells (Zhao et al., 2007) and it remains a possibility that these may play a part in the root hypoxia [Ca^2+^]_cyt_ signal with RBOH as an intermediary. Oxygen deprivation (and also sulfur and phosphate, Pi, deprivation) triggers programmed cell death (PCD) in mid-cortical cells for aerenchyma formation (Fagerstedt, 2010). This PCD is stimulated by ethylene and ROS and involves Ca^2+^ (Xu et al., 2013; Petrov et al., 2015). In wheat roots, anoxia causes mitochondria to release their Ca^2+^ and high [Ca^2+^]_cyt_ causes cytochrome c release (Virolainen et al., 2002). The abnormal mitochondrial ultrastructure in *Arabidopsis* caused by hypoxia is partially phenocopied by loss of GLR3.5 from the inner mitochondrial membrane, suggesting that this channel must be deactivated during PCD (Teardo et al., 2015). Further channels must now be identified to understand the hypoxic/anoxic response. Recent work on *Arabidopsis* roots has shown distinct, cell-specific levels of [Ca^2+^]_cyt_ after 24 h of hypoxia and highlighted the importance of CAX11 in controlling meristem [Ca^2+^]_cyt_ (Wang et al., 2016). Loss of CAX4 function resulted in lower tolerance of hypoxia thus further demonstrating the importance of Ca^2+^ sequestration in this stress response (Wang et al., 2016).

## Ca^2+^ Signaling in Nutrient Deprivation is an Emerging Area

Investigating [Ca^2+^]_cyt_ elevation in response to nutrient deprivation or resupply is technically challenging, particularly if using aequorin. Nevertheless it is now clear that nutrient levels can induce [Ca^2+^]_cyt_ changes and that downstream Ca^2+^ sensors regulate appropriate responses. The nutritional status of the root will have a part to play in determining the [Ca^2+^]_cyt_ signatures, particularly of the endodermis as the extent of suberization is set by nutrition (Barberon et al., 2016) and suberin lamellae determine endodermal [Ca^2+^]_cyt_ responses (Moore et al., 2002). To date, potassium, nitrate and boron have been studied.

### Potassium

Plants must maintain cytosolic K^+^ at around 80 mM for optimal growth even though soil concentration may be sub-millimolar and they deploy a multigene family of K^+^ transporters in homeostatic control (Shabala and Pottosin, 2014). As extracellular K^+^ decreases, the root epidermal PM hyperpolarizes and [Ca^2+^]_cyt_ increases (Demidchik et al., 2002). This increase can be abolished by gadolinium, implicating PM Ca^2+^ influx. The genetic identities of the PM Ca^2+^-permeable channels that would effect the [Ca^2+^]_cyt_ increase remain unknown. The hyperpolarised PM correlates with induction of *HAK5* expression in tomato root (Nieves-Cordones et al., 2008). *HAK5* encodes the PM High Affinity K^+^ transporter that facilitates K^+^ uptake from low external concentrations that are thermodynamically unfavorable for channel-mediated influx. In *Arabidopsis* roots, low K^+^-induction of *HAK5* expression (and other K^+^-deprivation genes) has been shown to depend on RBOHC activity and ROS (Shin and Schachtman, 2004). At present it is unclear whether the hyperpolarization of the PM directly activates RBOHC (which as an electron exporter may be voltage-dependent) or whether ROS-activated PM Ca^2+^ channels are involved in the K^+^-deprivation [Ca^2+^]_cyt_ signal. HAK5 activity in *Arabidopsis* roots is regulated by CIPK23-mediated phosphorylation, downstream of CBL1, CBL8, CBL9, and CBL10 (Ragel et al., 2015). The extent to which the transcriptional response to low K^+^ availability is governed by [Ca^2+^]_cyt_ is unclear but deficiency does result in upregulation of transcripts of Ca^2+^ signaling proteins (CaM, CBL, CIPK) in *Arabidopsis* seedlings (Armengaud et al., 2004) and sugarcane roots (Zeng et al., 2015). At higher external [K^+^], the AKT1 channel (Arabidopsis K^+^ Transporter1) facilitates uptake and its activity is promoted by CIPK23-mediated phosphorylation, downstream of CBL1 and CBL9 (Li et al., 2006; Cheong et al., 2007). This regulation is recapitulated in rice roots where CIPK23/CBL1 activate AKT1 (Li et al., 2014).

### Nitrate

Nitrate is the most important form of nitrogen for agriculture and deprivation triggers significant transcriptional and developmental responses. The effect of nitrate withdrawal on [Ca^2+^]_cyt_ has yet to be reported but recently it was shown that nitrate-starved *Arabidopsis* roots responded to nitrate resupply with a rapid, monophasic transient increase in [Ca^2+^]_cyt_ that was sensitive to lanthanides and phospholipase C (PLC) inhibition (Riveras et al., 2015). Lanthanum also blocked nitrate-induced InsP_3_ production, suggesting that Ca^2+^ influx across the PM activated a PLC. The [Ca^2+^]_cyt_ and InsP_3_ increases were entirely dependent on the PM nitrate influx transporter NRT1.1 (Nitrate Transporter1.1; Riveras et al., 2015). By using the *nrt1.1* mutant and pharmacological blockers, nitrate-induced gene transcription was also found to lie downstream of NRT1.1, and [Ca^2+^]_cyt_ elevation from PM influx and InsP_3_-gated store release. Calcium is also key to the regulation of nitrate uptake capacity as CIPK23, which is activated by CBL9 and CBL1, and dephosphorylated by ABI2 (a member of the PP2C protein phosphatase family; Léran et al., 2015,), phosphorylates NRT1.1 under low nitrate condition, thus converting it from a low to high affinity transporter (Ho et al., 2009). By contrast CIPK8 positively regulates the low-affinity phase of the nitrate primary response which includes transcriptional regulation, but its regulation of primary root elongation is concentration independent in *Arabidopsis* (Hu et al., 2009). CBL7, which is upregulated under nitrate deprivation, positively regulates the nitrate-dependent induction of *NTR2.4* and *NTR2.5* gene expression (Ma Q. et al., 2015). Given the lack of a [Ca^2+^]_cyt_ reporter line available in crops up until recently for rice, little is known about nitrate deficiency-induced [Ca^2+^]_cyt_ signaling but CaM protein abundance of wheat roots declines under nitrate deficiency, suggesting a remodeling of signaling systems (Jiang et al., 2015).

### Boron

Boron deficiency is widespread worldwide and particularly prevalent in China (Shorrocks, 1997). As B plays a dominant role in co-ordinating cell wall structure (Kobayashi et al., 1996), changes in cell wall stability are likely to influence the signal relayed into the cell upon B deprivation and indeed a rapid change in cell wall modulus has been observed (Goldbach et al., 2001). Early work in *Vicia faba* showed a release of membrane-bound Ca^2+^ into the apoplast (Mühling et al., 1998), raising the possibility of Ca^2+^ signaling during the early stages of B deficiency. Increased levels of both [Ca^2+^]_cyt_ and ROS have been suggested to lead to the increased root hair growth known to occur under B deprivation (González-Fontes et al., 2016). In cultured tobacco cells, transcriptional changes in response to short-term B deprivation (1 h) were abolished when withdrawing Ca^2+^ from the growth medium or upon treatment with the Ca^2+^ channel blocker lanthanum, thus implicating PM Ca^2+^ influx channels in generating a [Ca^2+^]_cyt_ signal (Koshiba et al., 2010). However, a transient [Ca^2+^]_cyt_ signal in direct response to B withdrawal has yet to be reported. Rather, work has focused on the possible remodeling of Ca^2+^ transport and signaling as a consequence of B deprivation.

Challenging cultured tobacco cells with Ca^2+^ resulted in a higher amplitude of [Ca^2+^]_cyt_ transient in B-deprived cells (1 h deprivation) than those grown under replete conditions (Koshiba et al., 2010). This suggests that B deprivation rapidly “resets” the PM’s Ca^2+^ transport systems to generate altered [Ca^2+^]_cyt_ responses. The [Ca^2+^]_cyt_ response of B-deprived cells was sensitive to lanthanum and diphenyleneiodonium, pointing to the involvement of PM Ca^2+^ channels and NADPH oxidases respectively (Koshiba et al., 2010). *Arabidopsis* roots expressing the YC3.6 [Ca^2+^]_cyt_ reporter exhibited higher levels of [Ca^2+^]_cyt_ at the apex than controls after 6 and 24 h of B deprivation (Quiles-Pando et al., 2013). This time course of B deprivation also resulted in significant upregulation of *CNGC19* (encoding a vacuolar channel), four genes of the ACA family of P_IIB_-type Ca^2+^-ATPases (*ACA1,10,12,13*) and *CAX3* encoding a vacuolar cation-H^+^ antiporter (Quiles-Pando et al., 2013). This suite of transporters could effect Ca^2+^ efflux from the vacuole (CNGC19) to increase [Ca^2+^]_cyt_ with clearance to the apoplast by ACA10-13 and sequestration to the vacuole by CAX3. How they are regulated remains to be determined, as does the involvement of the structurally compromised wall and the consequence of this higher level of apical [Ca^2+^]_cyt_. The area of higher [Ca^2+^]_cyt_ reported appears to correspond with the zone of inhibition of primary root elongation and the induction of cell death (Oiwa et al., 2013; Camacho-Cristobal et al., 2015). Once again, ethylene production appears to be upstream of NADPH oxidase-driven ROS production in growth arrest and death (Oiwa et al., 2013; Camacho-Cristobal et al., 2015).

Eight *CML* genes were also significantly upregulated after a day’s B deprivation of *Arabidopsis roots* (*CML11,12,23,24, 30.37,45.47*), as were three *CPK* genes (*CPK1,28,29*) all suggesting a distinct change in intracellular Ca^2+^ signaling (Quiles-Pando et al., 2013). This B deprivation also caused upregulation of *WRKY* transcription factors (TF) (*WRKY38,40,46*), two three *MYB* family TF (*MYB14,15,78*) and downregulation of two *BZIP* family TFs (*bZIP34,61*) (González-Fontes et al., 2016). B deprivation has also been found to promote the senescence-associated *WRKY6* TF in the root tip (Kasajima et al., 2010). It has been suggested that the *CML*s and *CPK*s upregulated by B deprivation are part of the chain of events leading to TF activation in the nucleus (González-Fontes et al., 2016) and this now requires direct testing. CIPK8 also positively regulates nitrate induced up-regulation of BOR1, a gene encoding a boron transporter responsible for xylem loading (Hu et al., 2009), suggesting that root signaling results in preservation of shoot B supply.

## Heavy Metal Stress has the Capacity to Distort Ca^2+^ Signaling

At the opposite end of nutritional deprivation is heavy metal stress. Industrial activity, mining and modern agricultural practices can lead to soil contamination by heavy metals (defined here as 7 g/cm^3^ and above). Although some of these metals (such as Zn, Cu) are required as micronutrients they can be damaging in excess whilst others (such as Cd) have no physiological role and can be deleterious even at low concentrations, often impairing mineral nutrition. The consequences of heavy metal exposure have been reviewed recently by Singh et al. (2016) and these authors explore signaling pathways (although not explicitly addressing Ca^2+^), intersects with hormonal responses and detoxification.

### Cadmium

Cadmium is a particular threat to Ca^2+^-based processes because of its similar size. A recent review by Chmielowska-Bak et al. (2014) summarized the effects of Cd on ROS accumulation, NO accumulation, MAP kinase activation and downstream responses in a wide range of plant systems and, importantly, did this as a function of Cd exposure. With regards to Ca^2+^ a key question is whether Cd (as a Ca^2+^ “substitute”) generates a signal in its own right or whether its impairment of Ca^2+^ homeostasis will be, in effect, the signal. Certainly Cd depolarises the rice root epidermal PM, which would impair Ca^2+^ influx and results in inhibition of root elongation (Li et al., 2012). Cd can permeate guard cell PM Ca^2+^ channels (Perfus-Barbeoch et al., 2002) and may compete with Ca^2+^ for entry into rice root hairs through PM HACC, thus limiting Ca^2+^ influx (Li et al., 2012). Competitive effects appear likely given the ability of exogenous Ca^2+^ addition to alleviate Cd inhibition of root growth of both terrestrial and aquatic plants, implying competition for uptake (Zhang et al., 2012; Rodriguez-Hernandez et al., 2015). Additionally, competitive inhibition by Cd of Ca^2+^ uptake through the wheat LCT1 transporter expressed in yeast has been observed (Clemens et al., 1998). Root hair growth in *Arabidopsis* is inhibited by Cd; it inhibits Ca^2+^ influx and so dissipates the apical [Ca^2+^]_cyt_ gradient needed for growth (Fan et al., 2011). This could reflect block of root hair Ca^2+^ influx channels or Cd outcompeting Ca^2+^ for that influx pathway. However, Yeh et al. (2007) observed an increase in rice root [Ca^2+^]_cyt_ following 15 min of Cd exposure, with a more tolerant variety sustaining a greater increase than a sensitive variety. In roots of the aquatic plant *Typha latifolia*, Cd exposure increases transcription of *TPC1* which suggests that vacuoles might release Ca^2+^ to the cytosol (Rodriguez-Hernandez et al., 2015). Inhibitor treatments indicated involvement of NADPH oxidases, hydroxyl radicals and CIPK upstream of a MAP kinase induction that linked to tolerance (Yeh et al., 2007). Cd stimulates expression and activity of NADPH oxidases in cucumber roots but it is not known if these responses are Ca^2+^-dependent (Jakubowska et al., 2015). It is worth noting that it is not only an increase in [Ca^2+^]_cyt_ that could stimulate NADPH oxidase activity but that also the restriction of Ca^2+^ entry to the cytosol could cause stimulation (Mortimer et al., 2008). It seems likely that in *Arabidopsis* roots it would be CAXs predominating in terminating a Cd-induced increase in [Ca^2+^]_cyt_. CAX4 mediates Cd and Ca^2+^ transport into *Arabidopsis thaliana* vacuoles and is needed for correct growth under Cd stress (Mei et al., 2009). *CAX1* expression is higher in roots of the Cd-tolerant *A. halleri* compared to the sensitive *A. thaliana* (Baliardini et al., 2015).

A key point for future studies is the intersect between Cd and hormones in relation to [Ca^2+^]_cyt_. Exogenous Ca^2+^ can ameliorate Cd’s inhibition of *Arabidopsis* root growth by counteracting effects of NO on auxin homeostasis (Hu et al., 2013; Li P. et al., 2016; Yuan and Huang, 2016). Cd also interferes with auxin homeostasis in barley roots (Zelinova et al., 2015). Auxin itself can increase *Arabidopsis* root [Ca^2+^]_cyt_ and this increase is mediated by CNGC14 at the PM, downstream of an unidentified auxin receptor (Shih et al., 2015). This begs the question of whether CNGC14 is an entry route for Cd and the pathway to disrupted auxin homeostasis. Additionally, Cd has been described as a “metallohormone” in that it triggers expression of brassinosteroid-regulated genes in *Arabidopsis* roots (Villiers et al., 2012). Brassinosteroids are themselves capable of transiently elevating [Ca^2+^]_cyt_ in *Arabidopsis* roots (through PM Ca^2+^ influx) and activate a possible DACC in wheat root PM (Straltsova et al., 2015). Again this raises the question of channel identity to help elucidate the relationship between Cd and brassinosteroid signaling and combat the effects of this potent soil contaminant.

### Copper, Gadolinium and Lead

In contrast to Cd, transition heavy metals could be capable of generating ROS directly and so perturb [Ca^2+^]_cyt_ signaling. Transition metals can catalyze production of hydroxyl radicals from superoxide anion and hydrogen peroxide through the Haber-Weiss reaction, with Cu^+^ and Fe^2+^ catalyzing hydroxyl radical production from hydrogen peroxide through the Fenton reaction (Richards et al., 2015). Unless levels of these catalytic metals are tightly controlled, production of hydroxyl radicals (the most potent of the ROS) could inflict significant oxidative damage. Taking Cu as the exemplar, it plays a positive role in hydroxyl radical-activated cell wall loosening for root elongation (Fry et al., 2002) and this could be coupled in *Arabidopsis* roots to hydroxyl radical-activation of PM Annexin1-mediated Ca^2+^ influx to stimulate exocytosis and growth (Foreman et al., 2003; Laohavisit et al., 2012). This model proposes regulation by extracellular hydroxyl radicals while Rodrigo-Moreno et al. (2013) have proposed that Cu^+^ binding at the intracellular face of the PM in *Arabidopsis* root tips catalyzes hydroxyl radical production to regulate ion flux. This would allow coupling of Cu transport into the root to regulation of PM Ca^2+^ influx channels. Certainly, low levels of Cu can promote elongation of *Arabidopsis* primary root and this effect is diminished by blocking PM Ca^2+^ influx channels (Demidchik et al., 2003). The effects of extracellular Cu on PM Ca^2+^ channels and therefore on [Ca^2+^]_cyt_ signaling are likely to be complex. Electrophysiological analysis of *Arabidopsis* root epidermal PM has shown that Cu not only activates Ca^2+^ channels through ROS production (Annexin 1; Laohavisit et al., 2012) but also blocks channels, the molecular identity of which remains unknown (Demidchik et al., 2003).

Catalytic production of ROS by Cu is not the only route to modulating [Ca^2+^]_cyt_. Longer-term exposure to Cu can stimulate exocytosis-mediated ROS production. Lin et al. (2013) found that inhibiting vesicle traffic with brefeldin also inhibited Cu-stimulated ROS production in rice roots. Whether this involved NADPH oxidase or other ROS generators remains to be determined. Rice root [Ca^2+^]_cyt_ increases in response to Cu addition and could link to NADPH oxidases and CIPK activity leading to MAP kinase activation (Yeh et al., 2007). It triggers oxidative stress in *Populus* roots that leads to regulation of CaM genes, implicating perturbation of [Ca^2+^]_cyt_ (Guerra et al., 2009).

In common with Cd, excess Cu also alters auxin homeostasis in roots and interferes with NO signaling (Lequeux et al., 2010; Kolbert et al., 2012). In excess, Cu stunts *Arabidopsis* root and root hair elongation and can inhibit lateral root outgrowth (Kolbert et al., 2012). Accumulation of lignin has been observed in both *Arabidopsis* and rice roots (Lequeux et al., 2010; Liu et al., 2015) and in the former accumulation is associated with the endodermis. It would now be interesting to ascertain whether such wall modification changes [Ca^2+^]_cyt_ signals in central cells or affects [Ca^2+^]_cyt_ propagative signaling to the shoot. Root tip cell death has also been observed (Huang et al., 2007; Lequeux et al., 2010; Rodrigo-Moreno et al., 2013). Cu-induced cell death in rice roots was attenuated by chelating extracellular Ca^2+^ thus implicating Ca^2+^ influx across the PM (Huang et al., 2007) while in *Arabidopsis* roots cell death was attenuated by addition of Gd^3+^ or verapamil as PM Ca^2+^ channel blockers (Rodrigo-Moreno et al., 2013).

Gd^3+^ is routinely used as a PM Ca^2+^ channel blocker, for example with proven efficacy against *Arabidopsis* root epidermal and root hair HACC, DACC and VICC (Véry and Davies, 2000; Demidchik et al., 2002; Miedema et al., 2008). It is also effective against the root PM hydroxyl radical-activated and H_2_O_2_-activated Ca^2+^ influx channels (Demidchik et al., 2003, 2007) and the Annexin1-mediated pathway (Laohavisit et al., 2012). As a consequence Gd^3+^ lowers Ca^2+^ influx (measured with ^45^Ca; Demidchik et al., 2002). From this it can be anticipated that Gd^3+^ would have the capacity to dampen stress-induced [Ca^2+^]_cyt_ signaling in roots, including that mediated by oxidative stress (Demidchik et al., 2003, 2007) and elicited by NaCl (Laohavisit et al., 2013). Pb is less well studied in terms of [Ca^2+^]_cyt_. Exogenous Ca^2+^ can protect against root Pb accumulation, suggesting competitive uptake (Rodriguez-Hernandez et al., 2015). However, Pb was found to evoke Ca^2+^ accumulation and diphenyleneiodinium-sensitive ROS increase in rice roots, the latter leading to MAPK activation (Huang and Huang, 2008). Entry of Pb into *Arabidopsis* roots involves CNGC1 as its deletion confers greater tolerance but whether Pb permeates this channel is unknown (Sunkar et al., 2000). Pb has also been shown to increase expression of *TPC1* in *Typha* roots (Rodriguez-Hernandez et al., 2015) but whether this results in increased [Ca^2+^]_cyt_ in this or other roots is not known yet.

## Mechanical Stress

Roots experience a range of mechanical stimuli, induced as they encounter soil particles or neighboring roots. An increase in [Ca^2+^]_cyt_ together with an apoplastic alkanisation are early effects induced by mechanical stimuli. One of the known downstream events from the increase in [Ca^2+^]_cyt_ is the upregulation of touch-sensitive genes such as *CML12* and *CML24* (Braam, 1992). Mechanically triggered [Ca^2+^]_cyt_ changes are dependent on the type of stimulus and the responding tissue (Legue et al., 1997; Monshausen et al., 2009). For example, manually bending an *Arabidopsis* root induces a rapid, biphasic increase in [Ca^2+^]_cyt_ on the convex side of the roots where cells are stretched (Monshausen et al., 2009) while previously compressed cells on the concave side of the roots show a monophasic and less intense increase in [Ca^2+^]_cyt_ upon return to their resting position. The source of Ca^2+^ is likely to be extracellular (Monshausen et al., 2009; Richter et al., 2009; Kurusu et al., 2012); with internal stores being mobilized to amplify the response (Chehab et al., 2009; Toyota and Gilroy, 2013).

Over-expressing *MCA1* in *Arabidopsis* lead to an enhanced [Ca^2+^]_cyt_ transient post mechanical stimulation by the addition of a membrane crenator, however, the *mca1* mutant showed no difference from the wild type (Nakagawa et al., 2007). Accordingly, Shih et al. (2014) showed no change in *mca1* apoplastic alkalinisation following root bending, which is closely related to the [Ca^2+^]_cyt_ signature. Thus some levels of compensation occur in mutants deficient in mechanosensitive Ca^2+^ channels. In contrast to the MCAs, MSLs (MscS-Like) were identified in *Arabidopsis* due to their sequence similarity to the bacterial Mechanosensitive channels of small conductance (MscS) (Haswell et al., 2008). These are encoded by a multiple gene family of 10 members, with MSL9 and MSL10 found in the PM of root cells and required for a mechanosensitive channel activity. They are predominantly anion channels and their relationship to mechano-stimulated [Ca^2+^]_cyt_ increase has yet to be shown but may be through PM voltage regulation. At this point, it is unclear whether these channels represent mechano-sensors or act downstream of yet unknown mechano-sensors.

Recently, the PM receptor-like kinase FERONIA has been implicated in regulating the *Arabidopsis* mechano-stimulated [Ca^2+^]_cyt_ increase and downstream transcriptional regulation (Shih et al., 2014). *feronia* mutants lack the second peak of the biphasic increase in [Ca^2+^]_cyt_ elicited in stretched cells by manual bending. However, the mechanism by which FERONIA can regulate [Ca^2+^]_cyt_ remains unclear as its kinase activity is not essential and targets are unknown. As far as we know, this study was the first in which a mutant with an aberrant mechano-stimulated [Ca^2+^]_cyt_ increase also showed a root skewing phenotype. Root skewing (deviation from the vertical when grown on vertical or inclined agar) may be influenced by mechano-sensing. At this point, it is unclear whether FERONIA also plays a role in the mechanical induction of lateral root formation (Richter et al., 2009).

### Cold Stress

Cold plays a key role in the regulation of physiology and development; the signaling processes relaying non-stressful temperatures (12°C and above) have been reviewed by Wigge (2013). The signaling cascades activated by cold stress (typically 4°C experimentally) and their relations with hormonal signaling have been reviewed by Knight and Knight (2012), Jeon and Kim (2013), Shi et al. (2015) and Eremina et al. (2016). Most studies on the effect of cold stress on [Ca^2+^]_cyt_ report on seedlings or leaves. From these it is well established that [Ca^2+^]_cyt_ elevation involves both Ca^2+^ influx across the PM and release from predominantly vacuolar stores (e.g., Knight et al., 1996; Mazars et al., 1997; Knight and Knight, 2000: Zhu et al., 2013). By using an extracellular Ca^2+^-reporting microelectrode, Sulaiman et al. (2012) confirmed that Ca^2+^ influx from the extracellular space is a component of the *Arabidopsis* root response to cold stress. For *Arabidopsis* roots, the [Ca^2+^]_cyt_ response to cold stress measured using aequorin is lower in amplitude and of much shorter duration than leaves (Zhu et al., 2013). Using aequorin Plieth et al. (1999) determined that the faster the rate of cooling *Arabidopsis* roots, the greater the [Ca^2+^]_cyt_ response. Sensitivity and magnitude of the [Ca^2+^]_cyt_ cooling response were enhanced by low temperature but repeated exposure to cold lead to desensitizing of the response. These results imply the existence of Ca^2+^ transport systems that could be regulated at the post-translational and possibly transcriptional levels. In *Arabidopsis*, cold-induced [Ca^2+^]_cyt_ elevation would activate a root PM calcium/calmodulin-regulated receptor-like kinase (CRLK1; Yang et al., 2010). This would then activate a specific mitogen-activated protein kinase kinase kinase (MEKK1) that then targets protein kinase kinase2 (MKK2; Furuya et al., 2013). This pathway leads to gene activation and freezing tolerance (Furuya et al., 2014). [Ca^2+^]_cyt_ elevation would also activate CIPK3, that acts an intersect with ABA signaling (Kim et al., 2003). Part of the transcriptional response to cold in *Arabidopsis* could be activated by increased [Ca^2+^]_cyt_ through CAMTA3 regulation of the transcriptional regulator CBF2 (C-Repeat/Dehydration Responsive Element Binding Factor2) (Doherty et al., 2009).

Cold stress has been found to depolarise the PM of root cells from cucumber and *Triana bogotensis* (Lyalin and Ktitorova, 1969; Minorsky and Spanswick, 1989), consistent with the effect of Ca^2+^ influx across the PM. A modeling exercise revealed the possible importance of a PM DACC in cold stress-induced [Ca^2+^]_cyt_ elevation of roots (White, 2009). Cold has been shown to activate a PM DACC in leaf protoplasts (Carpaneto et al., 2007) but this has not yet been shown for roots. As microtubule destabilization activates the *Arabidopsis* root PM DACC (Thion et al., 1998), this could link to the increased cold-induced [Ca^2+^]_cyt_ signal observed when microtubules are disrupted in *Nicotiana plumbaginifolia* leaf protoplasts (Mazars et al., 1997). Actin depolymerization is implicated in cold-induced Ca^2+^ influx across the PM into *Medicago sativa* suspension cells (Orvar et al., 2000). This in turn could relate to the cold-induced activation of pear pollen PM HACC that involves actin depolymerization (Wu et al., 2012). It would be timely therefore to investigate root PM for cold effects on HACC and DACC, exploring also the involvement of the cytoskeleton. In common with mechanical stress, cold stress could perturb the PM bilayer sufficiently to change Ca^2+^ channel activity. Work on *M. sativa* suspension cells showed that increasing membrane fluidity at 4°C prevented the Ca^2+^ influx across the PM that is necessary to trigger gene expression for freezing tolerance (Orvar et al., 2000). Conversely, imposing membrane rigidity at normal temperature triggers activation of the Ca^2+^-dependent MEKK1-MKK2-MPK4 pathway in *Arabidopsis* seedlings (Furuya et al., 2014). The possibility that mechanosensitive channels are involved in cold-induced [Ca^2+^]_cyt_ signaling merits investigation. However, recent work on rice roots indicates another route to Ca^2+^ influx. COLD1 has been identified as a PM activator of the heterotrimeric G protein subunit α (Ma Y. et al., 2015). It is required for cold-activated Ca^2+^ influx to roots (measured using extracellular Ca^2+^-reporting microelectrode) and elevation of root [Ca^2+^]_cyt_ (aequorin and cameleon determinations). This indicates that heterotrimeric G proteins lie upstream of PM Ca^2+^ channels in the cold response. In common with other stress responses, NADPH oxidase activity also appears in cold stress and needs to be placed in relation to Ca^2+^ channels. *Arabidopsis* roots exposed to cold stress use AtSRC2 (Soybean gene Regulated by Cold2) to enhance Ca^2+^ activation of the AtRBOHF NADPH Oxidase (Kawarazaki et al., 2013). This suggests that NADPH oxidase activity can lie downstream of Ca^2+^ channel activity. Curtailing cold-induced [Ca^2+^]_cyt_ elevation could involve vacuolar CAX1 activity in *Arabidopsis* (Catala et al., 2003).

## Conclusion and Future Prospects

A repeated message from this review is how incomplete our knowledge is of the channels mediating stress-induced [Ca^2+^]_cyt_ increases, and by extension those of organelles. Many members of channel gene families still await characterization. The identification of new families of channels is challenging and will require different approaches linking forward and reverse genetics to electrophysiology. Targets of Ca^2+^-binding and interacting proteins also require further study. Components common to different abiotic stresses are emerging such as *Arabidopsis* CIPK23 in K^+^ and nitrate deprivation. These common regulatory components are likely to represent critical steps where complex stress signals encountered in the soil are integrated in unified responses. Receptor like kinases such as FERONIA or PERK4 have emerged as new components in [Ca^2+^]_cyt_ signaling and perhaps other related proteins will be found to have a role in abiotic stress signaling. Remodeling of calcium signaling machinery after stress is also apparent with the possibility of components common to different stresses. For example, *Arabidopsis CNGC19* is upregulated under B limitation and salinity stress (Kugler et al., 2009). Finally, [Ca^2+^]_cyt_-dependent transcriptional responses can be delineated and future work could include the impact of stress-induced calcium signaling on epigenetic inheritance (Sani et al., 2013; Probst and Scheid, 2015).

## Author Contributions

All authors listed, have made substantial, direct and intellectual contribution to the work, and approved it for publication. KW, EM, SS, and JD wrote the review. KW produced the figures.

## Conflict of Interest Statement

The authors declare that the research was conducted in the absence of any commercial or financial relationships that could be construed as a potential conflict of interest.
